# In Vitro Investigation of the Effects of *Bacillus subtilis*-810B and *Bacillus licheniformis*-809A on the Rumen Fermentation and Microbiota

**DOI:** 10.3390/ani15040476

**Published:** 2025-02-07

**Authors:** Raphaële Gresse, Bruno Ieda Cappellozza, Didier Macheboeuf, Angélique Torrent, Jeanne Danon, Lena Capern, Dorthe Sandvang, Vincent Niderkorn, Giuseppe Copani, Evelyne Forano

**Affiliations:** 1 VetAgro Sup, UMR Herbivores, INRAE, Université Clermont Auvergne, 63122 Saint-Genes-Champanelle, Francevincent.niderkorn@inrae.fr (V.N.); 2 UMR 454 MEDIS, INRAE, Université Clermont Auvergne, 63122 Saint-Genes-Champanelle, Franceevelyne.forano@inrae.fr (E.F.); 3 Novonesis, 2970 Hørsholm, Denmark; brie@novonesis.com (B.I.C.);; 4 Department of Animal Nutrition and Feed Technology, Faculty of Animal Husbandry, Universitas Padjadjaran, Jatinangor, Sumedang 45363, West Java, Indonesia

**Keywords:** direct-fed microbials, ruminants, *Bacillus*, gut health, fermentation

## Abstract

Direct-fed microbials (DFMs) have shown the potential to improve livestock performance and overall health. Extensive research has been conducted to identify new DFMs and understand their mechanisms of action in the gut. *Bacillus* species are multifunctional spore-forming bacteria that exhibit resilience to harsh conditions, making them ideal candidates for applications in the feed industry and livestock production. This study investigates the mode of action of *B. licheniformis* and *B. subtilis* in the rumen using diverse in vitro techniques. Our results revealed that both strains germinated and grew in sterile rumen and intestinal contents from dairy cows and bulls. Gas composition analysis of in vitro cultures in a medium containing 40% rumen fluid demonstrated that germination of *B. licheniformis* and *B. subtilis* strains reduced oxygen levels, promoting an anaerobic environment favorable to rumen microbes. Enzymatic activity assays showed that *B. licheniformis* released sugars from complex substrates and purified polysaccharides in filtered rumen content. Additionally, the combination of *B. licheniformis* and *B. subtilis* survived and grew in the presence of a commercial monensin dose in rumen fluid media. The effects of *B. licheniformis* and *B. subtilis* on rumen fermentation activity and microbiota were studied using an in vitro batch fermentation assay. In fermenters that received a combination of *B. licheniformis* and *B. subtilis*, less CO_2_ was produced while dry matter degradation and CH_4_ production was comparable to the control condition, indicating better efficiency of dry matter utilization by the microbiota. The investigation of microbiota composition between supplemented and control fermenters showed no significant effect on alpha and beta diversity. However, the differential analysis highlighted changes in several taxa between the two conditions. Altogether, our data suggests that the administration of these strains of Bacillus could have a beneficial impact on rumen function, and consequently, on health and performance of ruminants.

## Simple Summary

The effects of two direct fed microbials strains, *B. licheniformis* 809A and *B. subtilis* 810B, on rumen fermentation parameters were investigated using various in vitro techniques. *B. licheniformis* 809A and *B. subtilis* 810B were able to germinate in rumen content, resist an in-feed monensin commercial dose, favor anaerobiosis, express enzymes, and beneficially impact in vitro rumen microbiota gas production and feed efficiency. These results indicate that *B. licheniformis* 809A and *B. subtilis* 810B display good potential for use as direct-fed microbials in ruminant production.

## 1. Introduction

Over the last decades, the use of direct fed microbials (DFMs) in livestock production has considerably increased. This type of feed additive aims to enhance overall animal health and performances while reducing reliance on antibiotics [1]. For DFMs to exert their intended effects, they must remain viable and active within the gastrointestinal tract (GIT) of the targeted animal species, as well as endure the challenges of feed production processes, such as pelleting, storage and manufacturing [2,3]. DFMs must also demonstrate resilience to in-feed antibiotics, such as monensin, which remains approved in several countries for livestock use. As spore-forming bacteria, Bacilli have the natural ability to turn into bacterial endospores containing a condensed and inactive chromosome and additional layers of coating [3]. This dormancy state is reached upon encountering difficult conditions or a decline in nutrients and gives *Bacillus* spp. the ability to resist extreme temperature or harsh intestinal conditions [3]. This characteristic represents a very attractive feature for animal nutrition as it limits the constraints of using protectants, encapsulating materials and cold transport chain [2,3]. Beyond their robustness, *Bacillus* spp. produce a diverse array of enzymes, such as xylanase, cellulase or amylase, which enhance feed digestibility and nutrient availability and improve animal performance [4,5]. Research in livestock has documented numerous benefits of *Bacillus* supplementation, such as reduction of adverse health events occurrence and mortality rates, improved weight gain and feed efficiency, and effective pathogen control and prevention [6,7].

In cattle, previous studies have shown that the administration of a *B. subtilis* natto strain could improve milk production and composition as well as increase the abundance of proteolytic and amylolytic bacteria in the rumen of dairy calves [8]. In a study from Smock et al. [9], daily administration of *B*. *subtilis* PB6 improved feed intake and performance in finishing cattle. In young ruminants, *B. subtilis* supplementation enhanced rumen development by increasing the surface area of rumen papillae [10]. Maternal supplementation of a *Bacillus*-based DFM increased prepartum body score condition and postweaning body weight gain and humoral response in their offspring [11].

Beyond performance enhancement, *Bacillus* species exhibit antipathogenic properties, which are critical as gastrointestinal pathogens impose significant economic and health burdens in animal production [12]. In vitro studies have demonstrated that *B. subtilis* limits the invasiveness of *Salmonella enterica* ser. Typhimurium in Caco-2 cells [13], while both *B. subtilis* and *licheniformis* strains already demonstrated in vivo protective properties against *S.* Typhimurium or *Clostridium perfringens* in challenged broilers [12,14]. Though those studies were conducted in poultry, such anti pathogenic properties against *Salmonella* species could also benefit cattle production, as cattle herds are considered to be a pathogen reservoir [15,16,17]. All together, these data suggest that *Bacillus* spp. would benefit cattle production, and further investigations are needed to unravel both their strain-dependent mode of action as well as potential other beneficial properties.

In this study, we used several in vitro techniques, such as classical microbiology, enzymatic activity assay and pathogen inhibition experiments, as well as the use of a batch fermentation model to study the impact and mode of action of *Bacillus subtilis* 810B and *Bacillus licheniformis* 809A (BOVACILLUS^TM^; Novonesis, Hørsholm, Denmark) used as a DFM in ruminants.

## 2. Materials and Methods

### 2.1. Origin and Sampling of Rumen and Digestive Content

For the growth curve experiment, rumen samples were collected from Holsteins cows slaughtered in the experimental slaughterhouse of INRAE “Herbipôle Experimental Unit” (UE 1414, National Research Institute for Agriculture, Food and Environment (NRAE), Saint-Genès-Champanelle, France). Digestive contents were obtained as described by Segura et al. [18] from eight 2-year-old healthy young bulls (*Bos taurus*) weighing 562 ± 26 kg and housed in the INRAE “Herbipôle Experimental Unit”. All animals were slaughtered in accordance with the guidelines of the local Ethics Committee and current INRAE ethical guidelines for animal welfare (Permit number: 63345001). All samples were rapidly collected and immediately brought to the laboratory. Rumen content was filtered through four layers of cheese cloth prior to being centrifuged twice at 10,000× *g* for 30 min to remove the endogenous microbiota. The clarified rumen content was stored at −20 °C until further use. Small intestine and colon contents were processed as described by Segura et al. [18]. Briefly, small intestine content was directly distributed in sterile tubes while colon contents were diluted 1:1 in reduced potassium phosphate buffer (50 mM potassium phosphate, resazurin 0.1%, 40 mM Na_2_CO_3_, 3 mM cysteine, pH 7.6), distributed in CO_2_ saturated Hungate tubes (Bellco Glass, Vineland, NJ, USA) and filter-sterilized (Millipore Steritop and Stericup system with a membrane pore size of 0.22 μm).

For the enzymatic activity assays and for batch fermentation, rumen content was collected from three dry Holstein cannulated cows housed at the INRAE Herbipôle unit (INRAE, Theix, France) fed ad libitum with grass and 500 g per day of concentrate feed. The concentrate feed contained 30% of beetroot pulp, 23% wheat, 20% barley, 15% rapeseed meal and 7.9% soybean meal (InVivo NSA, Chierry, France) (Table 1). The animals were adapted to the diet two weeks prior to the beginning of the first fermentation experiment and had free access to water. Fermentation experiments were conducted in three biological replicates using rumen content from one individual per replicate.

### 2.2. Strains, Media and Culture Conditions

*B. subtilis* 810B and *B. licheniformis* 809A (Novonesis, Hørsholm, Denmark) were weighted from spores’ bulk, diluted with peptone saline water (Maximum Recovery Diluent, Sigma-Aldrich, St. Louis, MO, USA) and submitted to a heat treatment at 80 °C for 10 min to remove vegetative cells. To evaluate their growth capacity in rumen and digestive contents, spores were inoculated into 96-well plates at a concentration of 5 × 10^6^ CFU/well in the following media: a rich medium containing 40% rumen fluid, pure rumen content with glucose, small intestine content, or colon content. Plates were sealed, placed at 39 °C for incubation and shaked every 20 min prior to optical density measurement. Growth was followed using a Tecan Spark^®^ plate reader (Tecan, Zurich, Switzerland). The rich medium containing 40% of clarified rumen juice was prepared following the instructions of Leedle and Hespell (1980) [19]. This rich medium contains a mix of sugars and starch as well as peptone and yeast extracts. Oxygen consumption capacity during germination was assessed using the rich medium containing 40% of clarified rumen fluid prepared using strict anaerobic techniques and dispensed in anaerobic Balch culture tubes with 100% ultra-pure CO_2_ gas. The experiment was conducted using 6 technical replicates. Spores were inoculated under CO_2_ atmosphere to the concentration of 10^8^ CFU/mL. Immediately after incubation, 3 mL of air was introduced into the Balch tubes using a syringe. Gas composition of the Balch tubes was measured right after inoculation and after 24 h of incubation (39 °C, 175 rpm) using a HP 6890 Gas Chromatograph (Agilent Technologies, Santa Clara, CA, USA) coupled with a TCD detector (Agilent Technologies, Santa Clara, CA, USA) and two series columns, Molecular Sieve 5A and Porapack Q (Agilent Technologies).

### 2.3. Compatibility with Monensin

To assess the compatibility of the *B. licheniformis* 810B and *B. subtilis* 809A corresponding to the commercial product BOVACILLUS™ (Novonesis, Hørsholm, Denmark), spores of both strains were diluted to the total concentration of 1.6 × 10^4^ CFU/mL in a 1:1 ratio in 96-well plates containing the 40% clarified rumen juice rich medium described above with or without a high commercial dose of monensin sodium salt (Sigma-Aldrich, Saint-Louis, MO, USA) corresponding to 10^7^ mg/animal per day based on a 200-L rumen volume. Growth of the strains were followed by optical density using a Tecan Spark^®^ plate reader (Tecan, Zurich, Switzerland). This experiment was repeated 3 times using independent 96-well plates, on which each condition was measured on 5 wells.

### 2.4. Enzymatic Activity Assay

Right after sampling, rumen content was filtered through four layers of cheese cloth prior to being centrifuged twice at 10,000× *g* for 30 min to remove the endogenous microbiota. The pH was adjusted to 6.8, and rumen content was distributed in bottles containing 2.5 g/L of one single substrate prior to heat-sterilization. Substrates used corresponded to the following purified substrates: arabinoxylan from wheat flour for reducing sugar assays (Megazyme, Wicklow, Irland), soluble starch from potatoes and carboxymethylcellulose (Sigma-Aldrich, Saint-Louis, MO, USA), or complex and unpurified substrates, such as flour from wheat, corn, soy and barley. Spores from both Bacillus strains were incubated at the concentration of 10^8^ CFU/ mL into sterile rumen content with a single substrate, either overnight or for 4 days at 39 °C under microaerobiosis (non-reduced medium) but incubation under CO_2_, as previously described in Bertin et al. [20] and Segura et al. [18]. After incubation, cultures were centrifuged at 10,000× *g* for 20 min and enzymatic activity assays were carried out on the supernatants, by measuring the reducing sugars released in the medium using the dinitrosalycilic acid method as described by Miller et al. [21]. The optical density of the samples was measured and compared to a standard curve made with glucose using a GenesysTM 30 spectrophotometer (Thermo Fisher Scientific, Waltham, MA, USA).

### 2.5. Pathogen Inhibition Assay

Ten grammes of autoclaved cattle feed was mixed with 40 mL of peptone saline water (Maximum Recovery Diluent, Sigma-Aldrich, St. Louis, MO, USA) and pre-inoculated or not with heat-treated spores of *B. licheniformis* 809A and *B. subtilis* 810B at the concentration of 5 × 10^5^ CFU/g of feed prior to aerobic incubation at 37 °C for 18 h. In parallel, *Salmonella enterica* Typhimurium ATCC14028 was pre-incubated on tryptic soy agar plates (Oxoid Holdings Limited, Altrimcham, UK). After pre-incubation, *S.* Typhimurium were added to the bottles of feed containing or not *B. licheniformis* and *B. subtilis* at the concentration of 1 × 10^5^ CFU/g of feed and incubated at 37 °C for 24 h. *S.* Typhimurium was enumerated at 0, 4, 8 and 24 h of incubation using MacConkey agar plates (Oxoid Holdings Limited, Altrimcham, UK) incubated for 18–24 h at 37 °C for both conditions.

### 2.6. Batch Fermentation

The experiment was authorized by the French Ministry for Research (No. 7138-2016092709177605-V5). Samples of rumen content were collected before the morning feeding from 3 ruminally cannulated cows housed in pasture and fed with grass and 500 g per day or concentrate.

In vitro fermentation was performed as previously described by Macheboeuf et al. [22]. Briefly, rumen content was filtered through an 800 μm polyester monofilament fabric which allows protozoa and fine particles carrying adherent bacteria to pass into the inoculum and mixed with an anaerobic buffer solution in a 1:2 ratio. Forty mL of this mixture was transferred into 120 mL serum bottles containing 600 mg of substrates grounded to 1-mm composed of corn silage (40%), grass silage (10%), hay (10%) and 40% of the concentrate feed detailed above. A total of 48 bottles were used per biological replicate split equally between 4 different treatments (*B. licheniformis*, *B. subtilis*, *B. licheniformis* + *B. subtilis* and control). The fermentation times were defined at 8 h to quantify starch degradation, and at 24 h, which represents the time between daily supplementations. Prior to the beginning of the fermentation, bottles were inoculated with a total of 5 × 10^7^ spores of either *B. licheniformis* 809A, *B. subtilis* 810B, or 1:1 ratio of both strains diluted in peptone saline water (Maximum Recovery Diluent, Sigma-Aldrich, St. Louis, MO, USA). Control flasks were inoculated with a similar volume of peptone saline water. Blank fermentation without substrates were performed at every fermentation run in order to calculate the net production of fermentation end-products. Bottles were previously flushed with N_2_ gas to eliminate the oxygen present inside. After inoculation, bottles were sealed with gas-tight rubber caps and incubated at 39 °C under intermittent agitation (600 rpm for 30 s followed by a resting period of 2.5 min) using magnetic stirrers. After 8 h and 24 h of incubation, the volume of gas produced was measured using a pressure transducer. A sample gas was collected using a 10 mL syringe for each bottle to further analyze gas composition. Five milliliters of fermentation media were aliquoted in a 15 mL Falcon tube and immediately stored at −80 °C for further microbiome analysis. The pH was measured, and the rest of the fermentation media were centrifuged at 3000× *g* for 5 min at 4 °C. Two times 1-mL of supernatant were mixed with 0.1 mL of orthophosphoric acid and stored at −20 °C for ammonia-N (NH_3_-N) analysis. For short-chain fatty acid (SCFA) downstream analysis, two aliquots of 800 μL of fermentation supernatant were mixed with 500 μL of deproteinization solution (1 g of crotonic acid and 5 g metaphosphoric acid, QSP 250 mL with HCL 0.5 N) and stored at −20 °C. Fermentation pellets were dried at 60 °C for 48 h, grounded to 1-mm and stored at room temperature for biochemical analysis.

### 2.7. Gas Composition, Biochemical, Ammonia-N and SCFA Analyses

Volume of gas produced during the fermentation was recorded after 3 h 30 min and 24h of fermentation using a pressure transducer [23]. Anaerobic conditions were checked by analyzing O_2_, and gas composition (CO_2_, CH_4_ and H_2_ produced during the fermentation process in the atmospheric phase of the bioreactors) was determined using a HP 6890 Gas Chromatograph (Agilent Technologies) coupled with a TCD detector (Agilent Technologies) and two series columns, Molecular Sieve 5A and Porapack Q (Aligent Technologies, Santa Clara, CA, USA). Ammonia-N was quantified as described by Weatherburn [24] using a Tecan Spark^®^ plate reader (Tecan, Zurich, Switzerland). The SCFAs were quantified in fermentation supernatants by gas chromatography using a PerkinElmer Clarus 580 gas chromatograph (Waltham, MA, USA). Neutral detergent fibers were determined as described by Van Soest et al. [25].

A mixed-model one-way ANOVA (lmer and ANOVA functions) with time point and bacterial treatment as fixed effects and fermentation replicate as a random effect was used to compare the concentration of the main SCFAs, gas, or NH3-N between the bacterial treatments using the R packages lme4 and car.

### 2.8. DNA Extraction from Batch Fermentation Samples

Total DNA was extracted from all samples using the Quick-DNA Fecal/Soil Microbe Miniprep Kit (Zymo Research, Irvine, CA, USA) according to the manufacturer’s instructions. The quality of the eluted DNA was assessed by agarose gel electrophoresis. Extracts were quantified using the Qubit dsDNA Broad Range Assay Kit (Invitrogen, Carlsbad, CA, USA) with a Qubit 2.0 Fluorometer (Invitrogen). Samples were stored at −20 °C prior to analyses.

### 2.9. MiSeq 16S rDNA Sequencing and Bioinformatic Analysis

The DNA concentration of all samples was measured using the Qubit dsDNA High Sensitivity Assay Kit (Invitrogen) with a Qubit 2.0 Fluorometer (Invitrogen) and diluted to 2 ng/μL prior to PCR amplification. The Bacterial V3–V4 region of 16S rDNA and the Archaeal 16S rDNA were, respectively, amplified with primers 357F 5′-CCTACGGGNGGCWGCAG-3′ [26] and 805R 5′-GACTACHVGGGTATCTAATCC-3′ [27] and primers 349F 5′-GYGCASCAGKCGMGAAW-3′ and 806R 5′-GGACTACVSGGGTATCTAAT -3′ [28]. Amplicons were generated using a Fluidigm Access Array followed by libraries construction and high-throughput sequencing on an Illumina MiSeq system (Illumina, San Diego, CA, USA) performed at the Carver Biotechnology Center of the University of Illinois (Urbana, IL, USA). The demultiplexed paired end Illumina sequence reads in the FastQ format were uploaded into the rANOMALY workflow [29]. During the rANOMALY pre-process, sequences were depleted of barcode, and the sequences with a non-appropriate length or containing ambiguous bases were removed. Next, sequences were denoised using the dada2 R package [30] and clustered into amplicon sequence variants (ASV) as a taxonomic unit. As described in the rANOMALY workflow [29], taxonomic assignments of ASVs were carried out by IDTAXA [31] using the Silva release 132 reference database [32]. Statistical and bioinformatic analyses were performed using the Rstudio software version 1.0. Visualization of data was performed using the ggplot2 R package version 3.2.1. Differential abundance between *Bacillus* treatment groups were determined using the Metacoder differential analysis tool [33]. All univariate statistical analyses were performed using linear mixed models (lme4 package version 1.1.21) with time point and bacterial treatment as fixed effects and fermentation replicate as a random effect. Analysis of variance tables was calculated with the car package (version 3.0.6). The means of each group were compared pairwise with the lsmeans package (version 2.30-0) with the Tukey correction. In all statistical analyses, only *p*-values below 0.05 were considered as significant.

## 3. Results

### 3.1. B. licheniformis and B. subtilis Can Germinate and Grow in Digestive Contents

**Figure 1:**
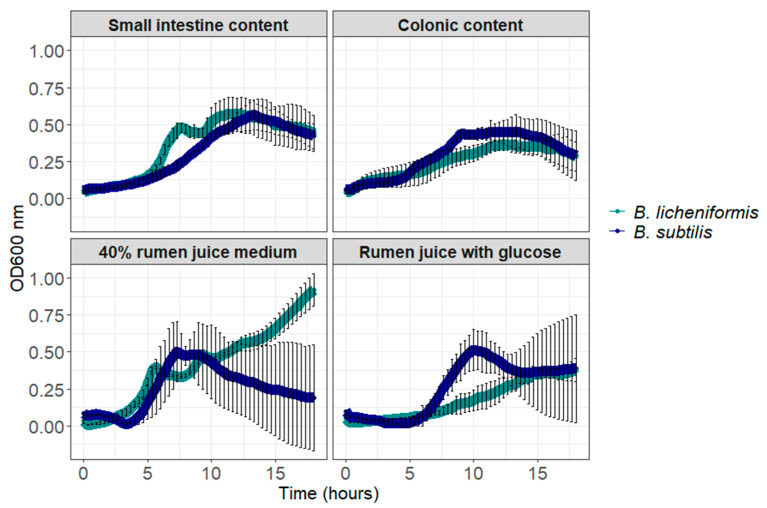
Growth of *B. subtilis* and *B. licheniformis* in digestive content, rumen juice and a rich medium containing 40% of rumen fluid (Mean OD 600 nm ± standard deviation).

*Bacillus licheniformis* reached an optical density (OD) of 0.92 ± 0.11 at time 18-h in the rich medium containing 40% rumen juice, mimicking the rumen environment (Figure 1). In rumen juice with glucose, small intestine content and colonic content, *B. licheniformis* reached its higher yield respectively at 18, 12 and 13 h of incubation corresponding respectively to maximum OD values of 0.38 ± 0.07, 0.57 ± 0.10 and 0.36 ± 0.04 (Figure 1). *Bacillus subtilis* reached rather similar yield in all media, corresponding to OD 0.50 ± 0.20 at time 7.3 h in rich medium, 0.51 ± 0.13 at time 10 h in rumen juice with glucose, 0.56 ± 0.10 at time 13.3 h in small intestine content and 0.45 ± 0.07 at time 11.3 h in colonic content (Figure 1).

### 3.2. Resistance to In-Feed Antibiotics

**Figure 2:**
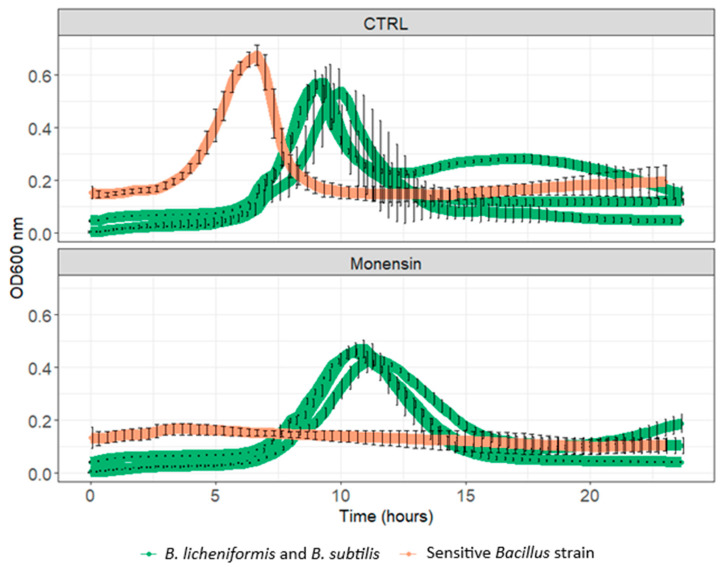
Co-incubation of *B. licheniformis* and *B. subtilis* (Bovacillus) with an in-feed commercial dose of monensin (“CTRL” = control condition without bacterial treatment) (Mean OD 600 nm ± standard deviation).

When incubated with a commercial dose of monensin in the 40% rumen containing rich medium, the co-incubation of *B. licheniformis* and *B. subtilis* reached a maximum optical density value of 0.40 ± 0.03 after 11 h of incubation, while the maximum optical density value in the control condition was reached after 9.3 h of incubation and corresponded to 0.57 ± 0.06 (Figure 2).

### 3.3. Growth and Germination of B. licheniformis and B. subtilis Favors Anaerobic Environment

**Figure 3:**
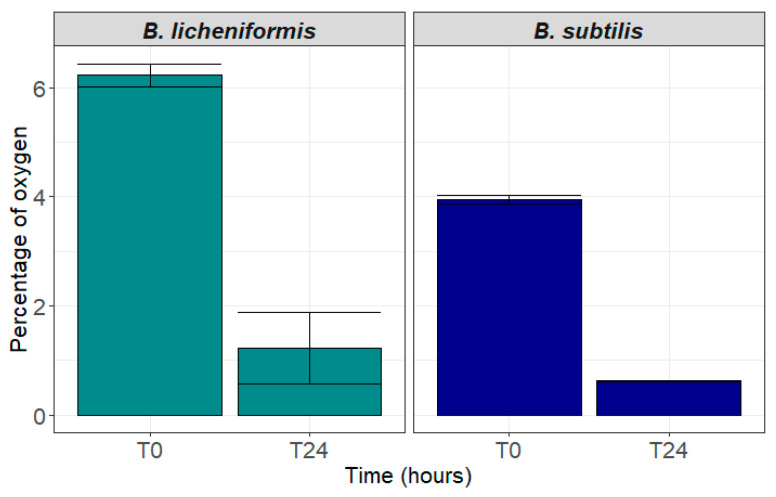
Oxygen percentage in the rumen-containing medium before inoculation (t = 0) and after 24 h incubation (t = 24) of spores of *B. licheniformis* (green bars) and *B. subtilis* (blue bars) (Mean percentage of oxygen ± standard deviation).

*B. licheniformis* and *B. subtilis* spores were respectively incubated with ~6% and ~4% of oxygen in Hungate anaerobic sterile tubes containing the rich 40% rumen juice medium (Figure 3). After 24 h of incubation, oxygen level was lowered down to ~0.6% and ~1.2% respectively for *B. subtilis* and *B. licheniformis* (Figure 3), indicating consumption of oxygen by the two strains after germination.

### 3.4. Enzymatic Capacity of B. licheniformis and B. subtilis in Rumen Content

**Figure 4:**
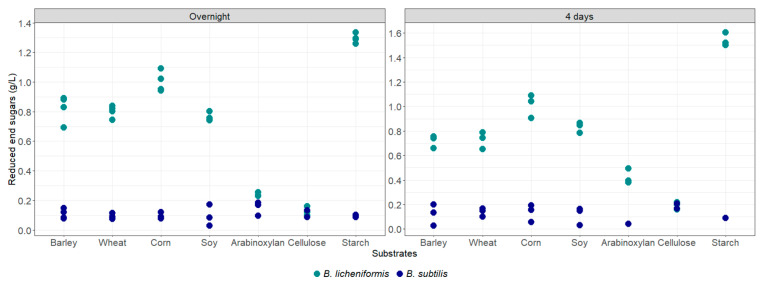
Concentration of released reducing sugars after consumption of complex and purified substrates by *Bacillus* strains cultivated in rich medium containing 40% rumen fluid.

After overnight and four-days incubation in sterilized rumen content with the various substrates, the degradation of barley, wheat, corn, arabinoxylan and starch by *B. licheniformis* and *B. subtilis* was assessed by quantification of the released reducing sugars (Figure 4). All the tested polysaccharides and complex substrates were efficiently degraded by *B. licheniformis* after 4 days, except cellulose, which was hardly degraded. The highest activity was measured on starch. Arabinoxylans were slightly degraded by this strain after overnight incubation, but they were clearly degraded after 4 days of incubation. On the other hand, *B. subtilis* enzymatic activities were limited under these conditions, as concentration of released reducing sugars stayed below 0.2 g/L even after 4 days of incubation (Figure 4).

### 3.5. Effects of Single Strains and Cocktails of B. licheniformis and B. subtilis on Rumen Batch Fermentation

**Figure 5:**
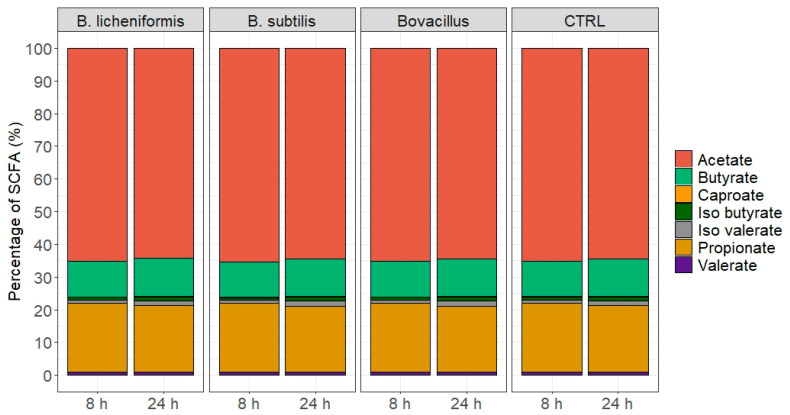
Short fatty acids relative abundance (%) produced by fermentation activity of the in vitro rumen microbiota in the control and *Bacillus* condition after 8 or 24 h of fermentation (“CTRL” = control condition without bacterial treatment).

The fermentation of the feed by the rumen microbiota in the fermenters led to a mixture of SCFA in the expected proportions for all conditions (Figure 5). No significant difference was observed between the control and the fermenters with the *Bacillus* strain after 8 h or 24 h of fermentation. No significant differences were highlighted in the pH and NH_3_ values at the end of both fermentation timepoints (Table 2 and Table 3).

### 3.6. Effects on Fiber Degradation

**Figure 6:**
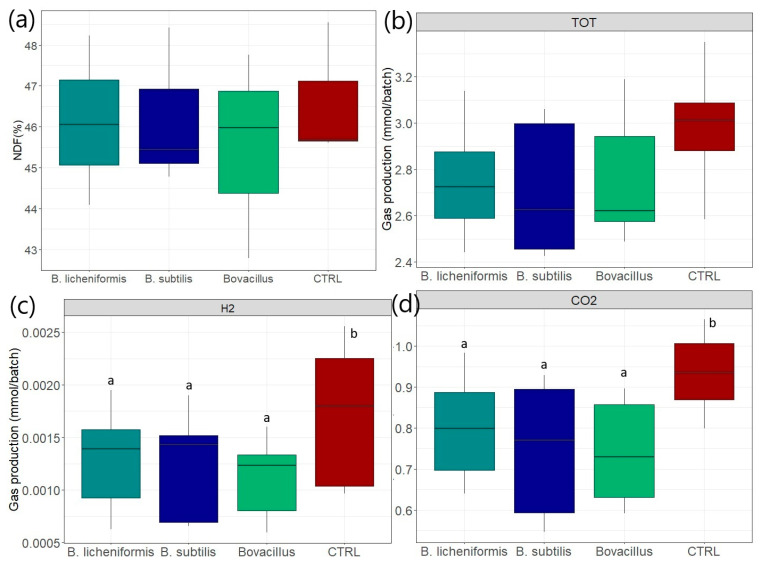
(**a**) Relative abundance of neutral detergent fibers (NDF) remaining in the fermentation media, (**b**) total “TOT” gas produced in the atmosphere of batch rumen fermenters after 8 h of fermentation, (**c**) hydrogen “H_2_” in the atmosphere of batch rumen fermenters after 8 h of fermentation, (**d**) carbon dioxide “CO_2_” in the atmosphere of batch rumen fermenters after 8 h of fermentation (“CTRL” = control condition without bacterial treatment). The conditions sharing the same letters are not significantly different from each other (*p* > 0.05).

After 8 h of fermentation, the percentage of NDF remaining in the fermentation media were not significantly different between groups and were respectively equal to 46.13 ± 2.07, 46.21 ± 1.94, 45.51 ± 2.52 and 46.62 ± 1.68% for the *B. licheniformis*, *B. subtilis*, *B. licheniformis* + *B. subtilis* and control conditions, respectively (Figure 6a). The percentage of NDF after the 24 h batch fermentations were similar and are presented in Supplementary Figure S1a.

### 3.7. Effects on Gas Production After 8 h

Total gas production was obtained in mmol/ batch. The quantity of CH_4_, CO_2_ and H_2_ produced was calculated from the percentages obtained from the gas chromatography analyses. The quantity of total gas, H_2_ and CO_2_ expressed in mmol/batch produced after 8 h of fermentation is presented in Figure 6b–d. After 8 h of fermentation, the total gas production had a tendency (*p* < 0.06) to be reduced in all *Bacillus* conditions compared to the control (Figure 6b). The percentage of H_2_ produced was significantly lower (*p* < 0.05) in batch fermenters supplemented with *Bacillus* compared to the control condition, with mean relative abundances of, respectively, 0.029 ± 0.012 and 0.041 ± 0.014% (Figure 6c). All the DFM-supplemented groups also produced significantly less CO_2_ than the control group (30.79 ± 2.17%), with mean relative abundances of, respectively, 28.64 ± 1.89, 27.62 ± 3.19 and 27.19 ± 2.79% for the *B. licheniformis*, *B. subtilis* and *B. licheniformis* + *B. subtilis* groups (Figure 6d). After 8 h of fermentation, the percentage of CH_4_ produced in the *B. licheniformis* group was significantly lower (*p* < 0.05) than in the control group, with mean relative abundances of, respectively, 7.20 ± 1.57 and 8.09 ± 0.72% (Supplementary Figure S1). The percentage of gas produced after 24 h is presented in Supplementary Figure S1b–d. Briefly, H_2_ production was significantly lower in the *B. licheniformis* + *B. subtilis* group compared to the other conditions. No other significant differences were observed for CH_4_ and CO_2_ after 24 h of fermentation (Supplementary Figure S1).

### 3.8. In Vitro Effects of B. licheniformis and B. subtilis on the Rumen Microbiota Composition

**Figure 7:**
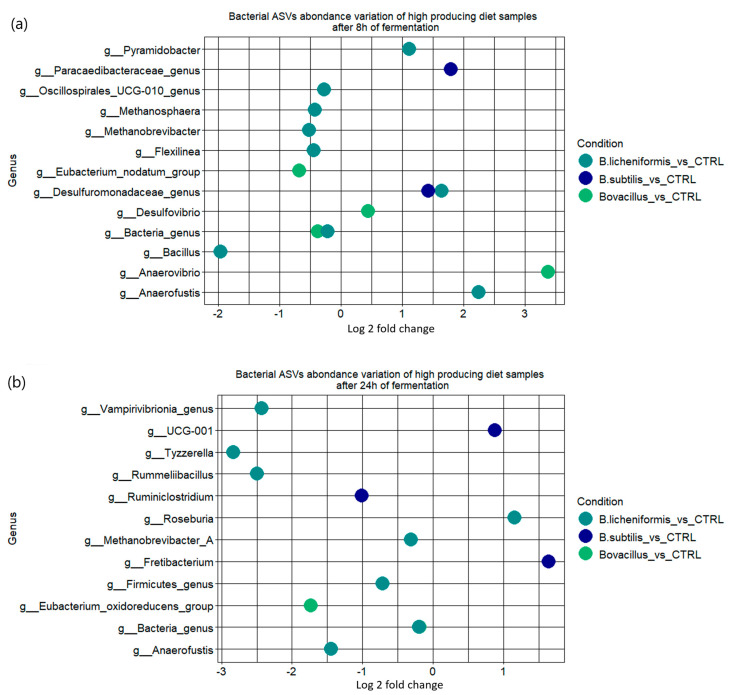
Significantly differentially abundant ASVs after 8 h (**a**) and 24 h (**b**) of fermentation in the *Bacillus* treatments compared to the control condition highlighted using the METACODER R package. “Bovacillus” corresponds to the cocktail of *B. subtilis* and *B. licheniformis.* Positive values relate to taxa more abundant in the *Bacillus* treatment while negative values relate to taxa more abundant in the control condition (“CTRL”).

The relative abondance of the principal phyla and genera for each treatment group is available in Table 4. No significant changes were observed in the principal phyla, families or genera in the in vitro rumen microbiota after 8 or 24h of fermentation. However, at the genus level, the *Bacillus* supplementation significantly affected the abundance of several other genera. For example, after 24 h of fermentation, the genera *Tyzzerella*, *Rummelibacillus* and *Methanobrevibacter* were significantly reduced in the bioreactor supplemented with *B. licheniformis* 809, while the genus *Fretibacterium* was significantly increased by *B. subtilis* 810 (Figure 7). The in vitro rumen microbiota alpha diversity indices were not significantly impacted by any of the treatments (Supplementary Figure S2).

### 3.9. Inhibition Capacity of B. licheniformis and B. subtilis on S. Typhimurium

**Figure 8:**
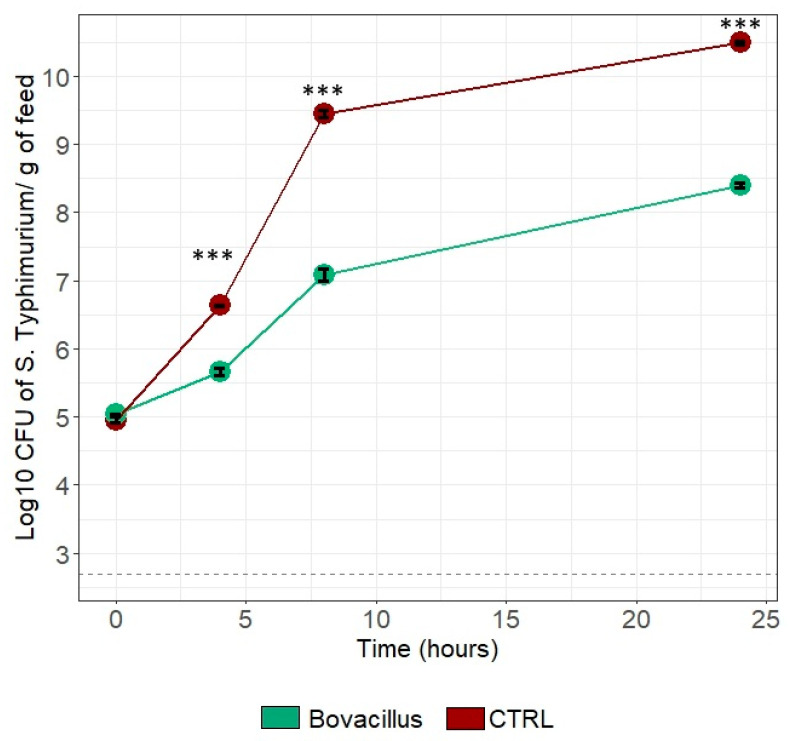
Count of *S*. Typhimurium after 24 h of co-incubation with a mixture of *B. licheniformis* and *B. subtilis* (Bovacillus) or alone as a control condition (“CTRL”). *** *p* value < 0.0001.

Co-incubation of *B. licheniformis* and *B. subtilis* with *S.* Typhimurium led to a significant decrease of *S.* Typhimurium CFU counts from 4 to 24 h of incubation. After 25 h of co-incubation, *B. licheniformis* + *B subtilis* was able to decrease *S.* Typhimurium growth by 2.12 log_10_ CFU/g of feed matrix, corresponding to a decrease of 27% (Figure 8).

## 4. Discussion

Direct fed microbials (DFMs) are by definition living microorganisms that, when administered in adequate amounts, confer benefits to their host [34]. This emphasizes the need to find strains which have the capacity to be metabolically active within their host GIT [2]. Stability, viability and preservation of DFM strains can be empowered via diverse strategies, such as encapsulation, drying technologies, or the use of protectants [2]. *Bacillus* spp. have the ability to be manufactured as spores, which considerably increases their resistance to pelletization, storage, transport and to the harsh environmental conditions of the GIT [35,36]. Moreover, DFMs may face additional challenges prior to reaching their site of action, including the presence of other feed additives, such as in-feed antibiotics. Monensin is a bacterial polyether ionophore widely used outside Europe in the feedlot industry [37]. In cattle, monensin supplementation reduces average feed intake while increasing weight gain by promoting propionic acid production in the rumen, thereby improving energy efficiency [38,39]. Monensin also mitigates the negative effects of high-grain diets in the rumen and decreases methane emissions, reducing the carbon footprint of beef and dairy products [39,40] while modifying the rumen microbiota composition, leading to a reduction in bacterial diversity [38,41,42]. Although ionophores have been banned in Europe as growth promoters, monensin is still widely used as a coccidiostat in poultry farming and as a preventive measure against ketosis in lactating cows [37]. As an antimicrobial, monensin inserts itself in the bacterial membrane, allowing rapid ion movements and causing drastic changes in intracellular ion concentration and pH [37]. Gram-positive bacteria are usually more sensitive to ionophores than Gram-negative species, but they can adapt over time [37]. In this study, we investigated the sensitivity *of B. licheniformis* 809A and *B. subtilis* 810B towards a commercial dose of monensin. The combination of these two strains exhibited a slight delay in growth and a modest reduction in yield when exposed to a commercial dose of monensin in a rumen fluid-containing medium. However, these in vitro results indicate that both strains remain active in the presence of monensin. Thus, administering monensin in cattle feed together with these *Bacillus* strains is unlikely to impair the strains’ beneficial effects on the rumen or intestinal microbiota. While Gram-negative bacteria are thought to resist ionophores due to their outer membrane, the adaptation mechanisms in Gram-positive bacteria are not well understood. Monensin-adapted Gram-positive cells exhibit altered cell wall characteristics, such as thickening of the cell wall or glycocalyx, which hinder ionophore binding. This adaptation appears to be a phenotypically expressed but genetically unstable trait, as it is typically reversible upon serial passage [43]. In feedlot cattle, the presence of antimicrobial resistance genes in the gut microbiota could not be correlated with the administration of monensin [38]. However, further studies are needed to elucidate the adaptation mechanisms in *Bacillus* species as well as the transmission of resistance properties across the rumen.

Rumen fluid displays a very specific and complex content including volatile fatty acids, ammonia, a high microbial biomass and many metabolite byproducts [44,45] which can considerably impact growth and activity on non-endogenous strains. It is, therefore, of interest to study the behavior of *Bacillus* spp. in conditions that resemble the rumen environment when selecting strains for ruminants. In this study, we successfully investigated the ability of spores from *B. licheniformis* and *B. subtilis* to grow, and consequently to germinate, in rumen fluid and digestive content. Germination of bacterial spores, as a result of increasing metabolic activity, have been linked to consumption of oxygen [46]. Anaerobiosis within the rumen is crucial for proper functioning of the inhabiting microbial community, which includes a wide variety of strict anaerobes [47]. A DFM having oxygen scavenging capacity can be thus of interest to favor the upkeep of rumen anaerobiosis. The scavenging capacity of *Bacillus* spp. has, to our knowledge, yet to be studied in rumen containing media, and our results indicate that both *B. subtilis* 810B and *B. licheniformis* 809A could have the ability to utilize the trace amounts of oxygen present in the rumen to germinate and grow.

On top of reaching their site of action in the gut and being able to turn into a metabolically active state, one of the most targeted modes of action to favor gut health and productivity in cattle would be benefiting feed digestibility. *Bacillus* spp. are recognized for producing a wide range of extracellular enzymes playing a significant role in digestion such as proteases, lipases, amylases, cellulases and xylanases [48,49,50]. Nonetheless, once produced, enzymatic activity can be influenced by many parameters such as pH, temperature, and the concentration of various molecules like MgCl_2_ or CaCl_2_ [51]. In this study, despite the widely known ability of *B. subtilis* to produce enzymes, only *B. licheniformis* 809A produced active enzymes able to degrade complex carbohydrates, starch or proteins from barley, wheat, corn and soy in pure rumen fluid buffered at pH 6.8. Moreover, DFMs can also impact feed digestibility in an indirect manner by influencing the rumen microbiota, and consequently, the endogenous enzymatic activity. In a study by Fuerniss et al. [52], the administration of a DFM containing *B. subtilis*, *B. licheniformis*, *B. amyloliquefaciens* and *B. pumilus* to commercial beef was shown to increase the proportion of fibrolytic bacteria in the cecum, thus favoring fiber digestion. In cannulated lactating dairy cows, *B. subtilis* and *B. licheniformis* mixture supplementation led to a higher Shannon alpha diversity index in the rumen microbiota, as well as a higher proportion of *Ruminococcaceae* and *Prevotellaceae* genera, among others [53]. Silva et al. [54] investigated the effects of a DFM containing *B. subtilis* and *B. licheniformis* on feed digestibility in cannulated steers and hypothesized that the observed increased acid detergent fiber digestibility could be due to higher efficient degradation of the hemicellulose fraction of the diet. Using in vitro rumen fermentation models can help dissect underlying modes of action of DFMs and has many advantages, such as reduced cost and time [55]. In this study, we used a rumen batch culture fermentation system to investigate the effects of *B. subtilis*, *B. licheniformis* and the combination of the two strains on the in vitro microbiota composition and activity, gas production and digestibility. In the rumen, gas production comes from the degradation of feed by the endogenous microbiota, and thus can reflect carbohydrate degradability and VFA production [56,57]. If no significant differences were highlighted for NDF digestibility in the present study, the same amount of fiber was degraded while less carbon dioxide was produced after 8 h of fermentation for all the *Bacillus* condition compared to control, which could be linked to a better feed degradation efficiency. While the major rumen microbial taxa were not modified by the presence of the two *Bacillus* strains, the relative abundance of several genera was modified, but except for the methanogenic archaea *Methanobrevibacter*, their function in the rumen is not known. Few other studies have explored the effects of the combination of *B. subtilis* and *B. licheniformis.* Pan et al. [58] highlighted a higher starch, dry matter and neutral detergent fiber digestibility, while Cappellozza et al. [59] reported only the two latest observations when the cocktail of *B. subtilis* and *B. licheniformis* was added in a rumen batch fermentation system. The lack of consistency observed between studies could be due to differences in the rumen fluid composition or to the use of different substrates to run the fermentation. Indeed, Cappellozza et al. [59] used commercial TMR in one of the studies, while Pan et al. [58] used various forages and concentrate feeds as fermentation substrates. Additionally, the diet received by the cows impacts rumen microbiota composition, and hence, its metabolic activity. The rumen microbiota comprises a liquid fraction, accounting for 20 to 30% of the total biomass, mainly including amylolytic and proteolytic bacteria, and a solid fraction, accounting for around 75% of the total microbial population and principally composed of fibrolytic bacteria [60,61]. Sampling for batch fermentation usually focusses on the liquid fraction of the rumen content filtrated through layers of gauze, and thus implies the elimination of part of the fibrolytic rumen microbiota, which could further explain the variety of observed results in the literature. If modes of action of *Bacillus*-containing DFMs are not yet to be fully elucidated, several in vivo studies have addressed their positive effects on feed digestibility in cattle. When using a multi-species DFM containing *B. subtilis* and *B. licheniformis* in beef steers, Miller et al. [62] reported an increase of body weight on day 28 and 55 of the experiment, and of dry matter intake and dry matter digestibility of rumen fluid, while Silva et al. [54] described an increase in acid detergent fiber digestibility. The effects of the supplementation by *B. subtilis* and *B. licheniformis* combination in growing steers receiving a high fiber diet led to an increase digestibility of dry matter and neutral detergent fiber as well as a higher fiber intake [63]. Single strains supplementation of *B. licheniformis* induced an increase of 8% of fiber degradation in Chinese Holstein cows [64]. All together, these results tend to confirm the positive effects of *B. subtilis* and *B. licheniformis* on rumen fermentation and feed degradability.

On top of performance, gut health, such as pathogen inhibition, is another targeted benefit of DFM supplementation in cattle. In the present study, an inhibition capacity of *B. licheniformis* and *B. subtilis* on *S.* Typhimurium was observed in vitro since the bacteria were able to reduce by more than 2 log the CFUs of the pathogen after 24 h of co-incubation. *Salmonella enterica* is an important zoonotic pathogen highly prevalent globally, and especially in ruminant production, as it is responsible for 93.8 million cases which result in about 155,000 human deaths annually [65,66,67]. In the present in vitro setting, we speculate that *Bacillus* spp. reduced *Salmonella* viability by various modes of action, including the secretion of antimicrobial compounds [68,69] or the reduction of pH [3,70]. The effects of a multi-species DFM containing *B. subtilis* and *B. licheniformis* have been also investigated in vivo against *S.* Typhimurium in challenged calves, and reduced pathogen shedding was observed in the fecal samples [71]. Further studies using epithelial cell culture models would be warranted to investigate other mode of action involving the host, such as limiting gut permeability or impairment of *Salmonella* virulence factors.

## 5. Conclusions

This study demonstrated that *Bacillus subtilis* and *B. licheniformis* had the potential to reach the rumen, their target site of actions, in a metabolically active state despite the specific composition of the rumen fluid or the use of in-feed antibiotics. *B. licheniformis* displayed interesting enzymatic properties while both *Bacillus* species showed some oxygen scavenging capacity. In a batch fermentation model, *B. subtilis* and *B. licheniformis* were able to impact gas production while keeping feed degradation properties of the rumen microbiota at the same level, which could induce a better utilization of the feed substrates. Altogether, our results indicate that the use of the combination of *B. subtilis* and *B. licheniformis* in ruminants could be beneficial to improve feed efficiency and gut health. Yet, further studies remain essential to better understand their effects and modes of action, particularly on in vivo trials.

## Figures and Tables

**Table 1:** Ingredients of the concentrate feed for the experimental units.

Ingredients	% of DM
Beet pulp	30.15
Wheat	20
Barley	20
Rapeseed	15
Soy	7.5
Corn	3
Molasses	1.5
Calcium phosphate	1
Vitamins and minerals	0.75
Salt	0.6
Magnesia	0.5

**Table 2:** Average pH values for each treatment group after 8 or 24 h of batch fermentation.

	pH at 8-h Fermentation	pH at 24-h Fermentation
*Bovacillus*	6.51 ± 0.12	6.16 ± 0.12
*B. Licheniformis*	6.49 ± 0.11	6.16 ± 0.13
*B. Subtilis*	6.51 ± 0.09	6.17 ± 0.13
Control	6.50 ± 0.11	6.17 ± 0.12

**Table 3:** Average concentration of NH_3_ for each treatment groups after 8 or 24 h of batch fermentation.

	NH_3_ (mmol/L) at 8-h Fermentation	NH_3_ (mmol/L) at 24-h Fermentation
*Bovacillus*	1.51 ± 0.87	13.35 ± 1.49
*B. Licheniformis*	1.69 ± 0.95	12.70 ± 1.10
*B. Subtilis*	1.65 ± 1.10	12.83 ± 1.53
Control	1.77 ± 0.81	13.16 ± 1.15

**Table 4:** Relative abundance of the main phyla and the main genera in the rumen in vitro microbiota after 8 and 24 h of batch fermentation (“Bovacillus” = cocktail of *B. licheniformis* and *B. subtilis*).

*Principal phyla at 8 h of Fermentation*	Bovacillus	*B. licheniformis*	*B. subtilis*	Control
*p__Firmicutes*	44.77	44.78	45.63	45.43
*p__Bacteroidota*	35.50	36.72	36.54	36.34
*p__Proteobacteria*	3.89	2.70	2.13	3.20
*p__Verrucomicrobiota*	3.74	3.82	3.73	3.61
*p__Actinobacteriota*	2.65	2.68	2.91	2.93
*p__Methanobacteriota*	1.48	1.90	1.70	1.40
*p__Fibrobacterota*	1.36	0.90	0.89	1.06
***Principal phyla at 24 h of Fermentation***	***Bovacillus***	***B. licheniformis***	***B. subtilis***	**Control**
*p__Firmicutes*	44.70	43.65	44.11	44.32
*p__Bacteroidota*	30.55	31.40	31.28	31.42
*p__Verrucomicrobiota*	8.39	8.67	8.44	8.60
*p__Actinobacteriota*	3.54	3.18	3.09	3.44
*p__Methanobacteriota*	2.22	2.39	2.06	1.97
*p__Proteobacteria*	2.22	2.35	2.46	2.05
*p__Fibrobacterota*	1.44	1.54	2.12	1.54
***Principal genera at 8 h of Fermentation***	***Bovacillus***	***B. licheniformis***	***B. subtilis***	**Control**
*g__Prevotella*	14.78	15.47	15.55	15.83
*g__Rikenellaceae_RC9_gut_group*	10.13	10.58	9.99	9.98
*g__Christensenellaceae_R-7_group*	8.18	8.54	8.85	8.71
*g__NK4A214_group*	4.14	4.60	4.57	4.41
*g__Clostridia_genus*	3.87	4.42	4.48	4.60
*g__F082_genus*	2.94	2.96	3.06	2.94
*g__WCHB1-41_genus*	2.86	2.94	2.81	2.81
*g__Ruminococcus*	3.00	2.74	2.87	2.78
*g__Olsenella*	2.23	2.23	2.40	2.47
***Principal genera at 24 h of Fermentation***	***Bovacillus***	***B. licheniformis***	***B. subtilis***	**Control**
*g__Prevotella*	9.78	10.10	10.02	10.59
*g__Rikenellaceae_RC9_gut_group*	9.65	9.76	9.57	9.38
*g__Christensenellaceae_R-7_group*	9.49	8.88	9.16	9.15
*g__WCHB1-41_genus*	6.55	6.85	6.83	6.64
*g__NK4A214_group*	5.20	5.12	5.11	5.05
*g__Clostridia_genus*	3.76	4.20	4.13	4.31
*g__F082_genus*	3.19	3.36	3.34	3.30
*g__Olsenella*	2.94	2.67	2.54	2.90
*g__Lachnospiraceae_NK3A20_group*	2.02	1.86	1.92	1.89

## Data Availability

The data generated and/or analyzed during the current study will be available in the BioProject database repository, https://www.ncbi.nlm.nih.gov/sra/PRJNA1186653 (accessed on 1 January 2025).

## Supplementary Materials

The following supporting information can be downloaded at: https://www.mdpi.com/article/10.3390/ani15040476/s1, Figure S1: Relative abundance of neutral detergent fibers (NDF) remaining in the fermentation media (a), methane (“CH4”) produced after 8 h of fermentation (b) and gas produced (c: total “TOT”; d: hydrogen “H_2_”; e: carbon dioxide “CO_2_”, f: methane “CH_4_”) in the atmosphere of batch rumen fermenters after 24 h of fermentation (“CTRL” = control condition without bacterial treatment); Figure S2: Alpha diversity indices of the rumen in vitro microbiota after 8 (a) and 24 (b) hours of batch fermentation (“Bovacillus” = cocktail of *B. licheniformis* and *B. subtilis*, “CTRL” = control condition without bacterial treatment).
