# Supplementary material for: In Vitro Investigation of the Effects of Bacillus subtilis-810B and Bacillus licheniformis-809A on the Rumen Fermentation and Microbiota
Source: Animals (Basel). 2025 Feb 7;15(4):476. doi: 10.3390/ani15040476 (PMC11851895; doi:10.3390/ani15040476)
Supplement: Supplementary file 1 [file animals-15-00476-s001.zip › animals-3346058-supplementary.pdf]

Supplementary Figures and tables

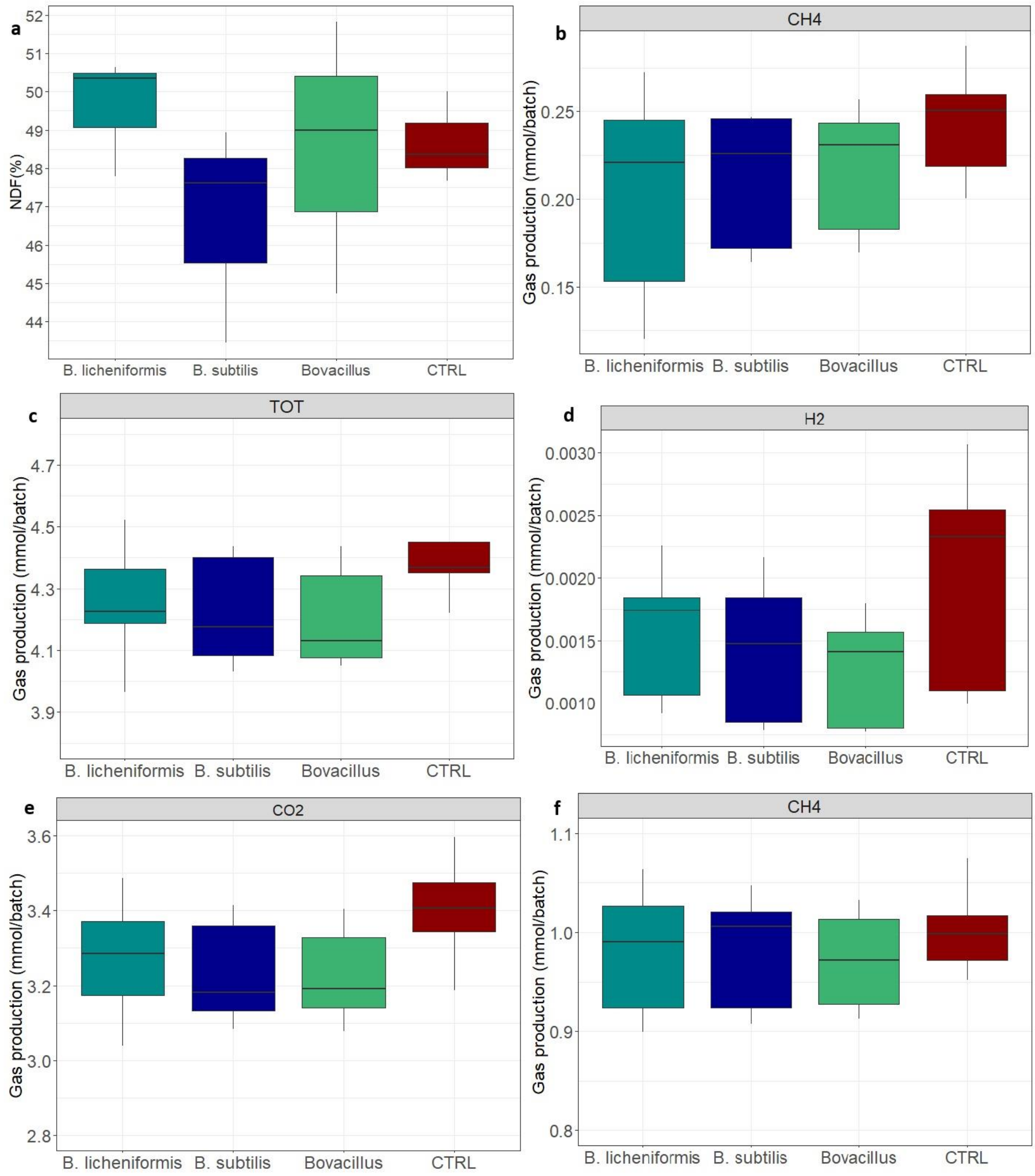

Supplementary Figure S1. Relative abundance of neutral detergent fibers (NDF) remaining in the fermentation media (a), methane ("CH4") produced after 8 hours of fermentation (b) and gas produced (c: total "TOT"; d: hydrogen "H2"; e: carbon dioxide "CO2", f: methane "CH4") in the atmosphere of batch rumen fermenters after 24 hours of fermentation ("CTRL" = control condition without bacterial treatment).

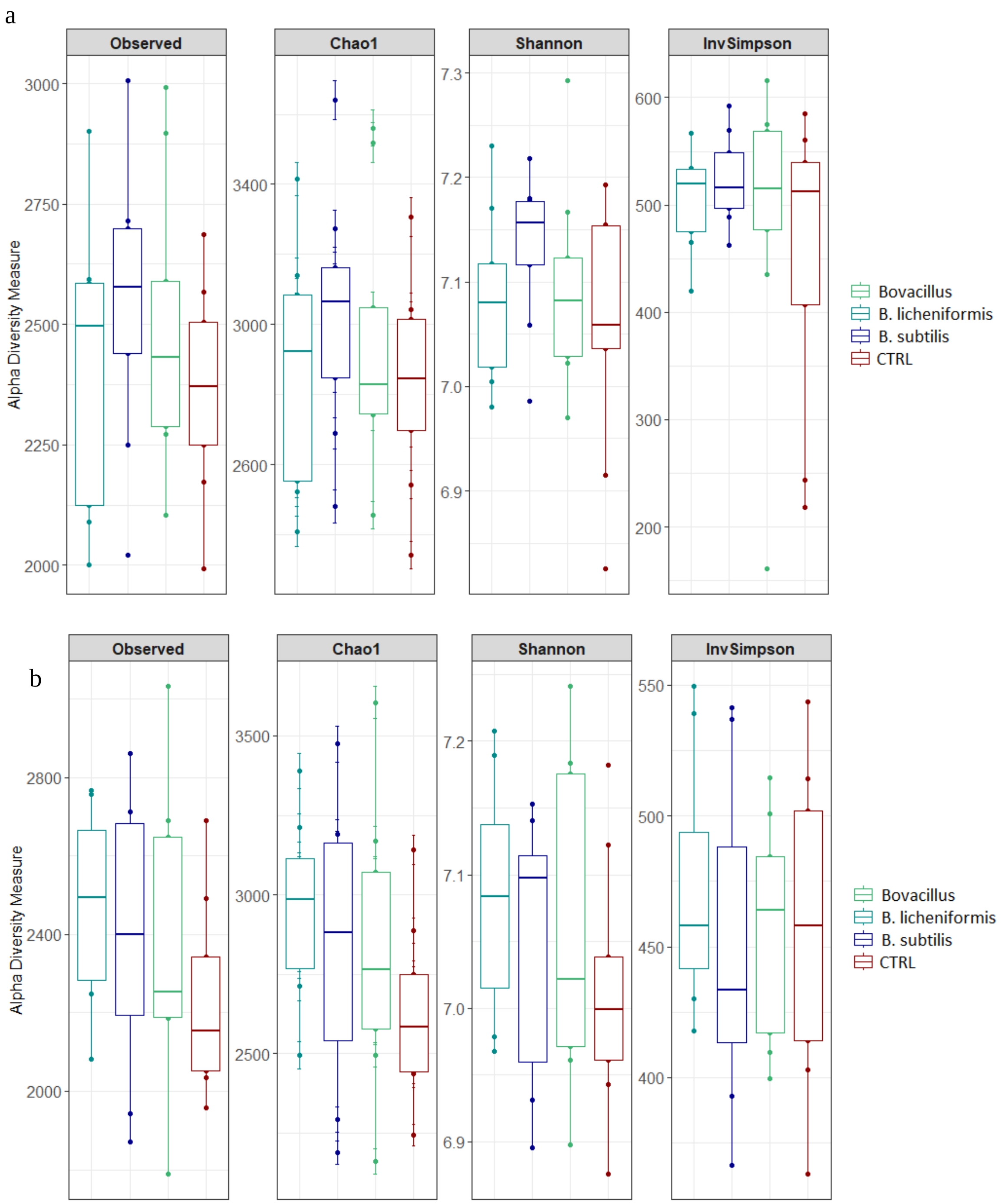

Supplementary Figure S2. Alpha diversity indices of the rumen *in vitro* microbiota after 8 (a) and 24 (b) hours of batch fermentation ("Bovacillus" = cocktail of *B. licheniformis* and *B. subtilis*, "CTRL" = control condition without bacterial treatment).
